# Duplication of the IGFBP-2 Gene in Teleost Fish: Protein Structure and Functionality Conservation and Gene Expression Divergence

**DOI:** 10.1371/journal.pone.0003926

**Published:** 2008-12-12

**Authors:** Jianfeng Zhou, Wenhong Li, Hiroyasu Kamei, Cunming Duan

**Affiliations:** 1 Department of Molecular, Cellular and Developmental Biology, University of Michigan, Ann Arbor, Michigan, United States of America; 2 Laboratory of Molecular Medicine, School of Medicine and Pharmacy, Ocean University of China, Qingdao, China; 3 Department of Aquaculture, Guangxi University, Nanning, China; Katholieke Universiteit Leuven, Belgium

## Abstract

**Background:**

Insulin-like growth factor binding protein-2 (IGFBP-2) is a secreted protein that binds and regulates IGF actions in controlling growth, development, reproduction, and aging. Elevated expression of IGFBP-2 is often associated with progression of many types of cancers.

**Methodology/Principal Findings:**

We report the identification and characterization of two IGFBP-2 genes in zebrafish and four other teleost fish. Comparative genomics and structural analyses suggest that they are co-orthologs of the human IGFBP-2 gene. Biochemical assays show that both zebrafish *igfbp-2a* and *-2b* encode secreted proteins that bind IGFs. These two genes exhibit distinct spatiotemporal expression patterns. During embryogenesis, IGFBP-2a mRNA is initially detected in the lens, then in the brain boundary vasculature, and subsequently becomes highly expressed in the liver. In the adult stage, liver has the highest levels of IGFBP-2a mRNA, followed by the brain. Low levels of IGFBP-2a mRNA were detected in muscle and in the gonad in male adults only. IGFBP-2b mRNA is detected initially in all tissues at low levels, but later becomes abundant in the liver. In adult males, IGFBP-2b mRNA is only detected in the liver. In adult females, it is also found in the gut, kidney, ovary, and muscle. To gain insights into how the IGFBP-2 genes may have evolved through partitioning of ancestral functions, functional and mechanistic studies were carried out. Expression of zebrafish IGFBP-2a and -2b caused significant decreases in the growth and developmental rates and their effects are comparable to that of human IGFBP-2. IGFBP-2 mutants with altered IGF binding-, RGD-, and heparin-binding sites were generated and their actions examined. While mutating the RGD and heparin binding sites had little effect, altering the IGF binding site abolished its biological activity.

**Conclusions/Significance:**

These results suggest that IGFBP-2 is a conserved regulatory protein and it inhibits growth and development primarily by binding to and inhibiting IGF actions *in vivo*. The duplicated IGFBP-2 genes may provide additional flexibility in the regulation of IGF activities.

## Introduction

Peptides of the insulin-like growth factor (IGF) and insulin family control growth, metabolism, reproduction, and longevity in a wide variety of animals ranging from invertebrates to humans. In extracellular fluids, these peptides are bound to and regulated by a family of IGF binding proteins (IGFBPs) and IGFBP related proteins [1], [2], [3]. Most IGFBPs are present in circulation, where they act to prolong the half-life of circulating IGFs. IGFBPs are also expressed in many peripheral tissues and are present in various biological fluids. In these local environments, IGFBPs regulate the availability of IGFs to the cell surface IGF receptors, thereby regulating the net biological activity of the IGF signaling pathway. Indeed, studies using a wide variety of mammalian cell culture systems have shown that IGFBPs have the ability to inhibit and/or potentiate the biological actions of IGFs, and some IGFBPs even exhibit ligand-independent biological activities *in vitro* [2], [4], [5].

There are six distinct IGFBPs, designated as IGFBP-1 to -6, and each is coded by a single copy gene in humans and other mammals [2], [4]. These IGFBPs all contain a highly conserved N-terminal domain, a conserved C-terminal domain, and a variable central linker (L) domain. The N-domain contains 12 conserved cysteine residues. C-domains have six conserved cysteine residues. These cysteine residues form intra-domain disulfide bonds [6], [7] and help to shape the globular structure of the N- and C-domains. The divergent L-domain often contains sites for post-translational regulation, including glycosylation, phosphorylation, and proteolysis. Despite similar domain arrangements, each IGFBP has distinct structural features. For example, IGFBP-2 has an arginine-glycine-glutamate (RGD) sequence in its C-domain and a heparin binding domain/motif (HBD) in its L domain. Comparative studies have shown that the gene, protein primary structure, and biological actions of IGFBPs are conserved in non-mammalian vertebrates [8]. In agreement with these structural features, both IGF- dependent and independent actions of IGFBP-2 has been reported in various mammalian culture systems, and RGD and/or heparin binding motifs have been implicated in the IGF-independent actions on cell growth and migration [9]. Ubiquitous expression of IGFBP-2 transgenes results in growth inhibition in mice [10], [11]. Although IGFBP-2 is the most abundantly expressed IGFBP in fetal tissues, the IGFBP-2 knockout mice were viable and fertile, and their prenatal and postnatal body weights were similar to those of their wild-type litter mates [12]. However, these mice had reduced spleen and enlarged liver. A more recent study indicates that the IGFBP-2 knockout mice had reduced trabecular bone volume and thickness in the adult male, but not female mice [13]. The modest phenotypes may be due to the compensatory adjustments in the expression of other IGFBPs and/or by maternal factors delivered through the placenta [12].

The use of the zebrafish has provided new insights into our understanding of the physiological functions of IGFBP-2 in vertebrate development [14], [15]. Targeted knockdown of zebrafish IGFBP-2a by antisense morpholino (MO) resulted in delayed development, reduced body growth, and disruptions to cardiovascular development [14].

Many teleost fish, including zebrafish, experienced an additional genome wide duplication event [16], [17]. Since then, while many of the duplicated genes have been lost, a substantial percentage of the duplicates have been retained. As a result, fish often have two co-orthologs in contrast to a single copy gene in humans and other mammals. One such co-orthologous pair is the zebrafish *igf-1r* genes [18], [19]. The retention of a particular pair of genes provided the unique opportunity to gain insights into the impact of evolution in reshaping genes and their roles in physiology. In this study, we have identified two IGFBP-2 genes in the zebrafish, medaka, fugu, tetraodon, and the stickleback genomes using a comparative genomics approach. Our molecular and functional analysis on the two zebrafish IGFBP-2 genes suggests that they have undergone subfunctionalization partitioning by evolving distinct gene expression patterns, while their protein functionality remains largely unchanged. Furthermore, taking advantage of the amenability of the zebrafish model, we investigated the relative contributions of the IGF binding domain, RGD, and HBD in IGFBP-2 actions *in vivo*.

## Results

### Zebrafish and other teleost fish have two IGFBP-2 genes

**Figure 1:**
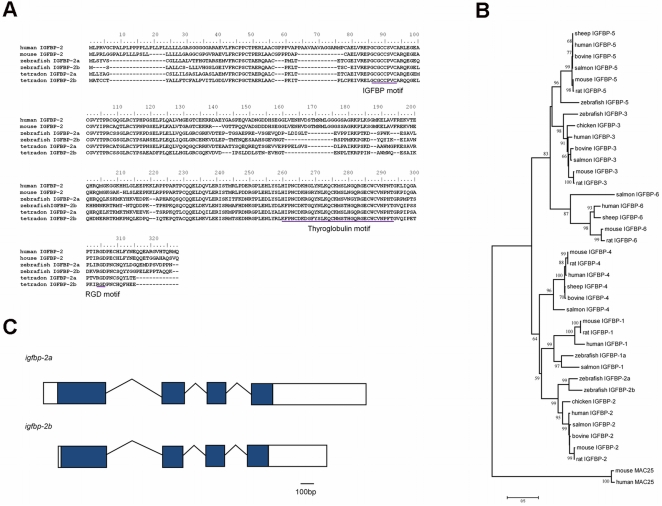
Structure of zebrafish IGFBP-2a and -2b. A) Amino acid sequence alignment of human IGFBP-2, mouse IGFBP-2, zebrafish IGFBP-2a and -2b, and tetradon IGFBP-2a and -2b. The IGFBP motif, thyroglobulin motif, and RGD motif are underlined. B) Phylogenetic analysis of the IGFBP gene family. Full-length sequences of IGFBPs were analyzed by MEGA4 with JTT matrix in neighbor-joining method. The reliability of each tree node was assessed by the bootstrap method with 1000 replications. Numbers on branches are percentage of times that the two clades branched as sisters. The clade with >50% bootstrap support was shown on each node. Human and mouse MAC25 genes were used as outgroups. The results indicate that the newly identified IGFBP is most closely related to members of the IGFBP-2 subgroup. Similar results were obtained using the Maximum Likelihood method and Maximum parsimony method. C) A schematic diagram showing the structure of zebrafish *igfbp-2a* and *igfbp-2b*. Filled boxes represent open reading frame, open boxes represent untranslated region (UTR), and lines represent introns.

In addition to the previously reported IGFBP-2 gene [20], we identified another IGFBP-2 like gene (ENSDARP00000019643) by searching the zebrafish genome. Using 5′- and 3′-RACE, its full-length cDNA was determined (EF507265). The encoded protein has a putative signal peptide of 17 amino acids (aa) and a mature protein of 248 aa with a predicted molecular mass of 29.7 kDa. Structural analysis suggests that there are 12 cysteine residues in its N-terminal domain (residues 1–79) and 6 cysteine residues in its C-terminal domain (residues 152–231) (Fig. 1A). A typical IGFBP motif is present in the N-domain and there is a thyroglobulin-1 motif in the C-domain. Among various members of the IGFBP family, the new IGFBP shares higher identities with IGFBP-2 than with other known IGFBPs. The overall sequence identities of this protein with the six human IGFBPs are 33% (IGFBP-1), 44% (IGFBP-2), 32% (IGFBP-3), 37% (IGFBP-4), 33% (IGFBP-5), and 30% (IGFBP-6). Its identities with the known zebrafish IGFBPs are 33% (IGFBP-1), 62% (IGFBP-2), 29% (IGFBP-3), and 31% (IGFBP-5), respectively. Human and other vertebrate IGFBP-2s contain an Arg-Gly-Asp (RGD) sequence in the C-terminal domain [1], [2]. An RGD motif is also present in the zebrafish IGFBP at a.a.position 226 to 228. The putative heparin binding motif (PKKXRP) located in the central domain in all the known mammalian IGFBP-2 is absent in the zebrafish protein. Phylogenetic analysis using full-length amino acid sequences of known vertebrate IGFBPs grouped the newly identified IGFBP into the IGFBP-2 clade (Fig. 1B), indicating that it is a co-ortholog of human IGFBP-2. We therefore named the previously reported zebrafish IGFBP-2 gene [20] as *igfbp-2a*, and this new gene as *igfbp-2b*.

**Figure 2:**
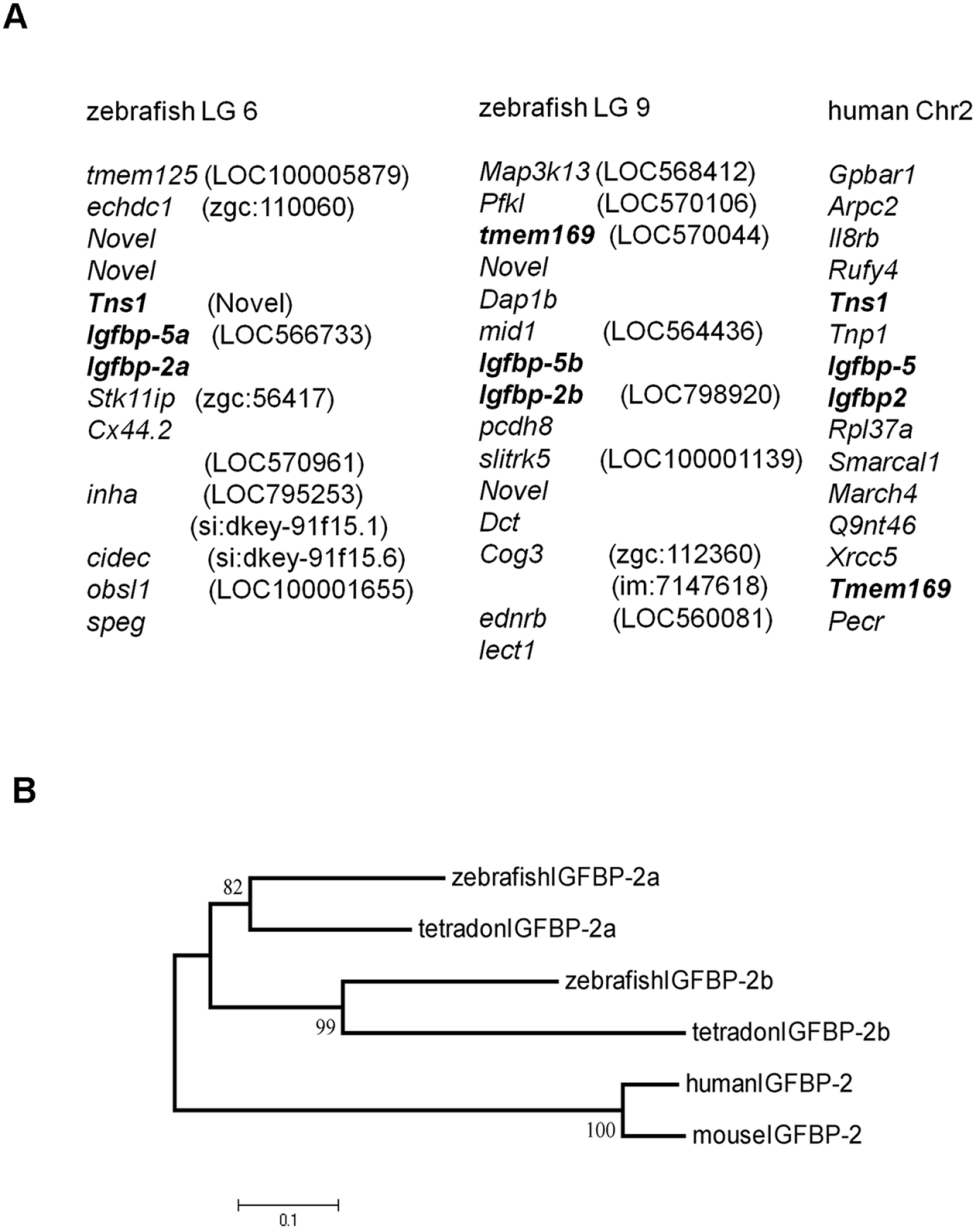
Synteny and phylogenetic analysis of zebrafish IGFBPs. A) Conserved synteny between human and zebrafish IGFBP-2 genes. Zebrafish *igfbp-2a* and *igfbp-2b* are located on linkage group (LG) 6 and 9, and human *IGFBP-2* is located on chromosome 2. The human *IGFBP-2* and *IGFBP-5* are neighboring genes in a tail-to-tail orientation. Likewise, zebrafish *igfbp-2a/igfbp-5a* and *igfbp-2b*/*igfbp5-b* are also neighboring genes in a tail-to-tail orientation. B) Phylogenetic tree of IGFBP-2. Amino acid sequences of full-length IGFBP-2s were analyzed. Values on branches are percentages that the two clades branched as sisters (1000 run). The results indicate that teleost *igfbp-2a* and *igfbp-2b* are likely a result of a fish-specific genome duplication event.

The genomic structure of *igfbp-2a* and *igfbp-2b* was determined by searching the zebrafish genome database. *igfbp-2a* is 30,665 bp long and contains 4 exons and 3 introns (Fig. 1C). The size and overall structure of *igfbp-2b* are similar (Fig. 1C). The chromosomal loci of *igfbp-2a* and *igfbp-2b* were also mapped. While *igfbp-2a* is located on linkage group LG 6, *igfbp-2b* is mapped to LG 9 (Fig. 2A). The human IGFBP-2 gene resides on chromosome 2 adjacent to the IGFBP-5 gene in a tail-to-tail fashion [21]. In the zebrafish genome, we found that zebrafish *igfbp-2a* and *igfbp-5a* are located on LG 6, next to each other in a tail-to-tail fashion. Likewise, zebrafish *igfbp-2b* and *igfbp-5b* are next to each other in a tail-to-tail fashion. In the proximate regions, there are several other zebrafish genes (*tns1*, *tmem169*) whose orthologs are located on human chromosome 2 (Fig. 2A). This conserved synteny strongly suggests that *igfbp-2a* and *igfbp-2b* are co-orthologs of the human IGFBP-2 gene.

To determine whether the duplication of the IGFBP-2 gene is unique to zebrafish or a more general phenomenon in teleost fish, we searched the available genome databases for medaka (*Oryzias latipes*), Fugu (*Takifugu rubripes*), Tetraodon (*Takifugu nigroviridis*), and the three-spine stickleback (*Gasterosteus aculeatus*) and investigated the evolutionary relationships within this subfamily. As in the case of zebrafish, there are two igfbp-2 genes in all four teleoest species and their primary structures are highly similar to that of IGFBP-2 (Fig. 1A). Phylogenetic analysis using mammalian IGFBP-2 as an out-group indicated that the duplication of the igfbp-2a/b subfamily expanded during the ray-fin fish genome duplication (Fig. 2B).

### Zebrafish *igfbp-2b* encodes a secreted protein that binds IGFs

**Figure 3:**
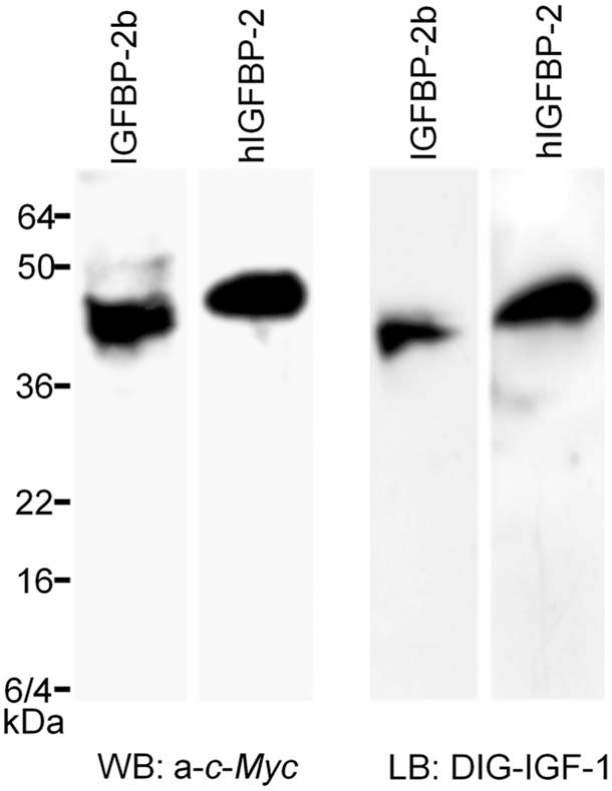
Zebrafish *igfbp-2b* encodes a secreted protein that binds IGFs. Conditioned media was prepared from HEK293T cells transfected with pCMV-human IGFBP-2-Myc (hIGFBP-2) or pCMV-zebrafish IGFBP-2b-Myc plasmids. The conditioned media were analyzed by Western immunoblotting (WB) using c-Myc antibody (left panel) and ligand blotting using DIG-labeled human IGF-1 (right panel).

Previously, we have shown that zebrafish IGFBP-2a binds IGFs with high affinity and specificity [20]. To determine whether *igfbp-2b* also encodes a functional IGFBP, we constructed an expression plasmid by subcloning the zebrafish IGFBP-2b ORF into the pcDNA3.1/ Myc-His(-)A expression vector. As a positive control, a pcDNA3.1/Myc-His(-)A-human IGFBP-2 plasmid was also generated. After these plasmids were introduced into HEK293T cells by transient transfection, conditioned media were prepared and subjected to Western immunoblot and ligand blot analysis. Both human and zebrafish IGFBP-2b had apparent sizes between 36 and 50 kDa on SDS-PAGE, likely due to the Myc tagging and posttranslational modifications. Zebrafish IGFBP-2b had a slightly smaller apparent size than human IGFBP-2 (Fig. 3A). Ligand blotting showed that both zebrafish IGFBP-2b and human IGFBP-2 proteins bind human IGF-1 (Fig. 3B). These results suggest that zebrafish *igfbp-2b* also encodes a secreted protein that binds to IGFs.

### Zebrafish *igfbp-2a* and *-2b* exhibit distinct spatiotemporal expression patterns

**Figure 4:**
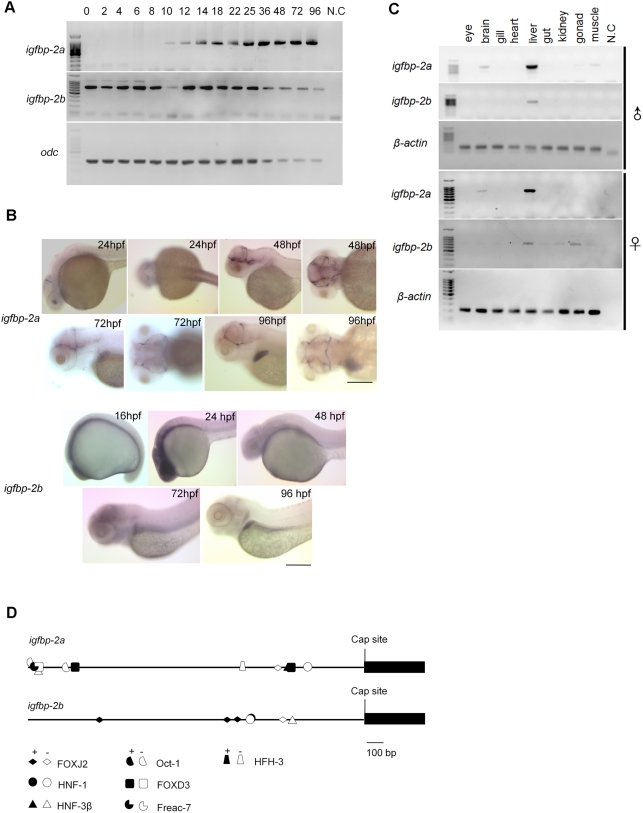
Temporal and spatial expression patterns of *igfbp-2a* and *igfbp-2b*. A) RT-PCR analysis result. The developmental stages are shown at the top, hpf, hour post fertilization. N.C., negative control. odc, (ornithine decarboxylase). B) *In situ* hybridization analysis of whole mounted embryos. Embryos of indicated stages were analyzed. Scale bar = 100 µm. C) Tissue distribution of *igfbp-2a* and *igfbp-2b* mRNA in male and female adult fish. D) Schematic diagram comparing the 5′-flanking region of *igfbp-2a* and *igfbp-2b* 2,000 bp before the cap site are shown. Close symbols indicate DNA binding elements in forward orientation and open symbols indicate those in reverse orientation.

The spatial and temporal expression patterns of *igfbp-2a* and *-2b* were determined by semi-quantitative RT-PCR and *in situ* hybridization. As shown in Fig. 4A, IGFBP-2a mRNA was not detectable until 10 hpf. The IGFBP-2a mRNA levels gradually increased from 10 to 36 hpf and was maintained at high levels thereafter. Likewise, IGFBP-2a mRNA was undetectable by in situ hybridization in early embryonic stages. At 24 hpf, IGFBP-2a mRNA was detected in the lens and cranial region (Fig. 4B). At 48 and 72 hpf, IGFBP-2a mRNA was detected in the brain boundary vasculature. Expression in these regions persisted throughout the hatching period (Fig. 4B). By 96 hpf, IGFBP-2a mRNA expression was most abundant in the liver. This dynamic spatiotemporal pattern of igfbp-2a is in good agreement with a previous study [14]. As shown in Fig. 4A, IGFBP-2b mRNA was easily detected by RT-PCR throughout embryogenesis. In agreement with the RT-PCR data, *in situ* hybridization analysis showed that IGFBP-2b mRNA was detectable at all stages examined. In early embryos, the IGFBP-2b signal was detected in most, if not all, tissues at low levels. At 96 hpf, IGFBP-2b mRNA expression was mostly detected in the liver (Fig. 4B).

In the adult stage, the highest levels of IGFBP-2a mRNA were found in the liver in both the male and the female (Fig. 4C). In addition, modest levels of IGFBP-2a mRNA were seen in the brain. Low levels of IGFBP-2a mRNA were also detected in the muscle and gonad in male, but not female adult fish. There appeared to be sexual dimorphism in the expression pattern of IGFBP-2b mRNA in the adult stage. In the male, it was exclusively expressed in the liver. In the female fish, it was not only detected in the liver, but also in a number of other tissues, including the gut, kidney, ovary, and muscle. These results indicate that the duplicated *igfbp-2a* and *-2b* have evolved distinct spatial and temporal expression patterns.

To determine if the two zebrafish IGFBP-2 genes have associated with divergent regulatory elements, we analyzed a 2 kb fragment upstream of the cap site in *igfbp-2a* and *igfbp-2b*. Transcription factor binding sites within the regions were predicted by the on-line program Match using a library of mononucleotide weight matrices from TRANSFAC 6.0 (www.gene-regulation.com) with both core similarity and matrix similarity of the transcription factor binding sites set higher than 0.85. The results revealed that the two promoter regions have different regulatory element sites (Fig 4D).

### Ectopic expression of IGFBP-2a and IGFBP-2b causes a similar degree of reduction in embryonic growth and development but has no effect on cell fate and patterning

**Figure 5:**
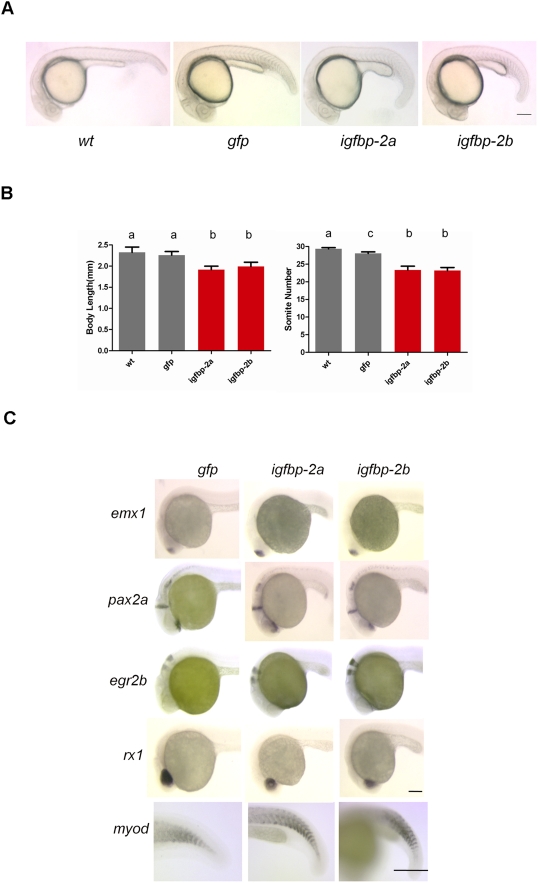
Ectopic expression of IGFBP-2a and IGFBP-2b causes a similar degree of reduction in embryonic growth and development but has no effect on cell fate and patterning. A) Representative images of wild-type, GFP mRNA (800 pg/embryo), IGFBP-2a mRNA (800 pg/embryo), or IGFBP-2b mRNA 800 pg/embryo) injected embryos at 24 hpf. B) Body length and somite number of the groups indicated. Results are from three independent microinjection experiments. The total embryo number for each group is 79 (wt), 95(*gfp*), 79(*igfbp-2a*), and 100 (*igfbp-2b*). Values are represented as means±S.E. (n = 3). Groups with common letters are not significantly different from each other (p<0.05). C) Whole mount *in situ* hybridization analysis of various marker genes in *gfp* mRNA-injected control (left column), *igfbp-2a* mRNA-injected (central column), and *igfbp-2b* mRNA-injected (right column) embryos at 24 hpf: *emx1* expression in the forebrain; *pax2a* expression in the optic stalk, mid-hindbrain boundary, and hindbrain; *egr2b* expression in the third and fifth rhombomeres of the hindbrain; *rx1* expression in the retina; *myoD* expression in the somatic myotome. Scale bar = 100 µm. Similar patterns were observed in all embryos examined in each group (n = 8–14).

To determine and compare the biological activities of zebrafish IGFBP-2a and -2b, we performed *in vivo* functional studies. For this, capped mRNAs encoding zebrafish IGFBP-2a and -2b were generated and introduced into zebrafish embryos by microinjection. As shown in Fig. 5A, embryos injected with either zebrafish IGFBP-2a or -2b were morphologically normal, but they were smaller in size and developmentally delayed compared to GFP mRNA injected or wild type control embryos. The body lengths of IGFBP-2a and IGFBP-2b injected embryos were 1.92±0.14 mm and 1.99±0.17 mm at 24hpf, which are significantly smaller than the 2.32±0.22 mm and 2.26±0.15 mm of the WT and GFP-injected embryos (p<0.05). At 24 hpf, wild type embryos and GFP mRNA injected control embryos had 29.4±0.6 and 28.0±0.8 somites, respectively. In comparison, embryos injected with either zebrafish IGFBP-2a or IGFBP-2b had only 23.4±1.9 and 23.2±1.5 somites (p<0.05) (Fig 5B). They were developmentally equivalent to wild type or GFP-injected embryos at ∼20–21 hpf. We next performed *in situ* hybridization to determine any potential defects in patterning. Overexpression of either IGFBP-2a or IGFBP-2b did not alter the brain and somite patterning, as indicated by mRNA expression patterns of *emx1* (labeling forebrain), *rx1* (retina), *pax2a* (optic stalk, mid-hindbrain boundary, hindbrain), *egr2b* (third and fifth rhombomeres), and myoD (somite) (Fig 5C). These data suggest that the two IGFBP-2 isoforms have comparable biological activities. When expressed, they both inhibited embryonic growth and developmental rates without notable effects on cell fate determination or patterning.

### The conserved growth inhibitory action of IGFBP-2 is dependent on IGF binding but not its interaction with heparin or integrin *in vivo*

**Figure 6:**
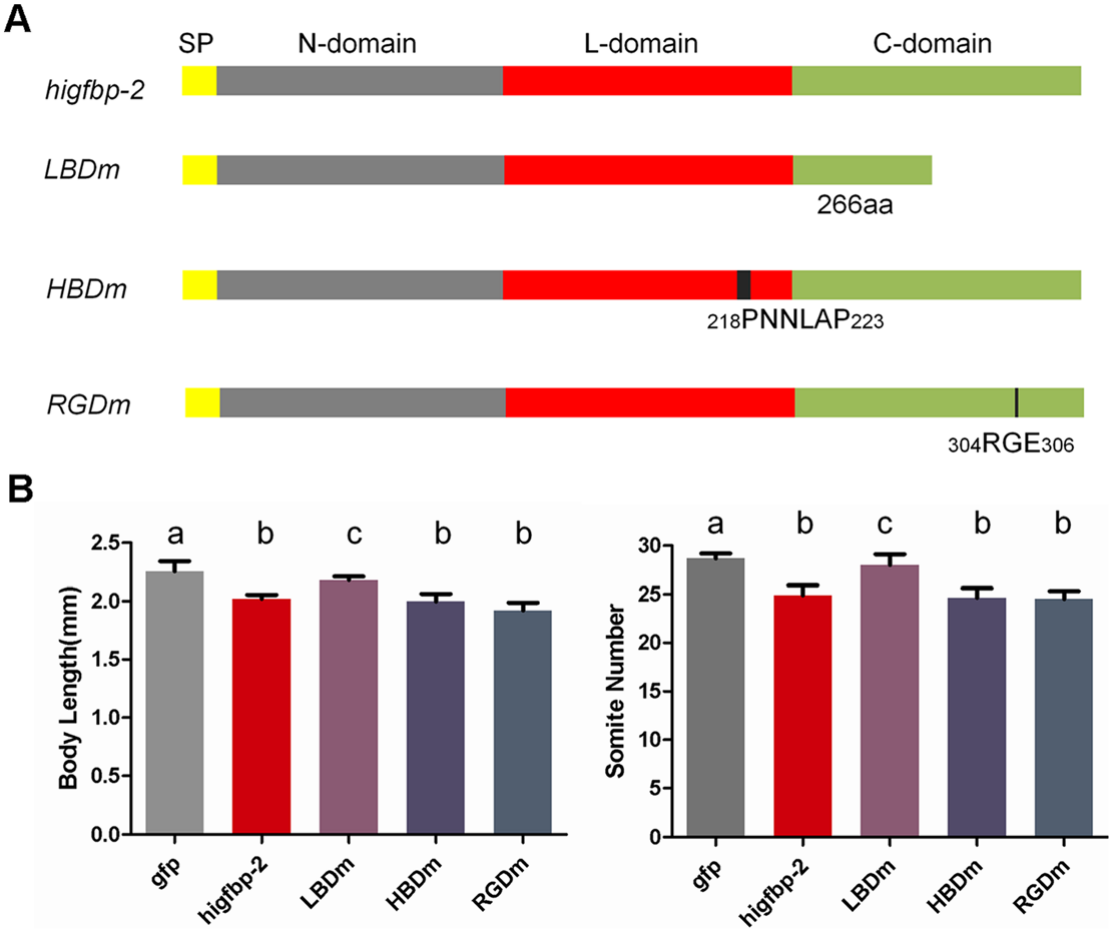
The growth inhibitory effect of IGFBP-2 is evolutionarily conserved and is dependent on its IGF binding but not its interaction with heparin or integrin. A) A schematic diagram showing the native and mutant IGFBP-2 structure. The ligand binding mutant (LBDm) was engineered following Forbes et al. [6], heparin binding domain mutant (HBDm) following Russo et al. [22], and RGD mutant (RGDm) following Wang et al. [23]. B) Effects of expression of proteins shown in A) on embryo growth and developmental rate in vivo. Capped mRNA (800 pg/embryo) was injected into 1–2 cell stage embryos. The embryos were raised to 24 hpf and body length and somite number were determined. Results are from three independent microinjection experiments. The total embryo number for each group is 91(*gfp*), 94 (*higfbp2*), 131 (*LBDm*), 79 (*HBDm*), and 81(*RGDm*). Values are represented as Means±S.E. (n = 3). Groups with common letters are not significantly different from each other (p<0.05).

To determine whether the biological action of IGFBP-2 is conserved between teleost and humans, capped mRNAs encoding human IGFBP-2 were generated and introduced into zebrafish embryos. Embryos injected with human IGFBP-2 mRNA phenocopied those of zebrafish IGFBP-2a and -2b mRNA injected embryos, i.e., they were smaller, developmentally delayed, but morphologically normal. Further quantitative analysis showed that ectopic expression of human IGFBP-2 caused a comparable decrease in growth and developmental rates (Fig. 6B).

In addition to its ability to bind IGFs, human IGFBP-2 was reported to also act independently of IGF via its RGD and/or heparin binding motifs in cultured mammalian cells [22], [23]. The *in vivo* significance of these *in vitro* findings, however, has not been well studied. Because the biological action of IGFBP-2 is conserved, we tested the functional importance of these motifs *in vivo* by taking advantage of the zebrafish model. For this, human IGFBP-2 mutants with altered ligand binding site (LBDm), RGD (RGDm), and heparin-binding site (HBDm) were engineered (Fig. 6A). The effects of these mutations on IGF binding and cell surface association have been characterized *in vitro* by others [6], [22], [23]. The corresponding capped mRNA was synthesized and introduced into zebrafish embryos. While the RGDm and HBDm had similar effects in slowing down growth and developmental rates (Fig. 6B), embryos injected with LBDm mRNA were indistinguishable from those of GFP mRNA injected controls in morphology, body size, and somite number (Fig. 6B), suggesting that altering the IGF binding site abolishes its biological activity. These results suggest that IGFBP-2 is a conserved regulatory protein and it acts by binding to IGF and inhibiting its actions *in vivo*.

## Discussion

In this study, we have demonstrated that the ortholog of tetrapod IGFBP-2 has duplicated in the zebrafish and other teleost fish. The conclusion that there are two IGFBP-2 genes in these teleost genomes is supported by phylogenetic and detailed structural data. Fish IGFBP-2a and -2b share much higher identities with human IGFBP-2 than with other human IGFBPs. All known vertebrate IGFBP-2s contain an RGD sequence in the C-terminal domain[2]. An RGD motif is also present in the zebrafish IGFBP-2a and -2b. Phylogenetic analyses grouped the newly identified genes into the IGFBP-2 clade. The strongest evidence came from the conserved synteny analysis. In humans, IGFBP-2 and -5 genes are located next to each other on the same chromosome in a tail-to-tail orientation [1]. In this study, we found that *igfbp-2a* and *igfbp-5a* are neighboring genes located on LG6 in a tail-to-tail orientation, resembling the situation of human IGFBP-2 and -5 genes localized on chromosome 2. If *igfbp-2a* and *igfbp-5a* arose by a chromosomal duplication event, one would expect that *igfbp-2b* should also be located next to another *igfbp-5b* gene. Indeed, our analysis shows that *igfbp-2b* and *igfbp-5b* genes are neighboring genes on LG9. In addition to these IGFBP genes, we were able to identify other orthologous genes between zebrafish LG9 and human Chr.2. This conserved synteny strongly argues that zebrafish *igfbp-2a* and *igfbp-2b* are co-orthologs of the human IGFBP-2 gene.

The fact that there are also two IGFBP-2 genes in the medaka, fugu, tetraodon, and the stickleback implies that the origin of this gene duplication is ancient, likely before the divergence of ray-fin fish and tetrapods. This notion is also supported by the phylogenetic analysis data and is in good agreement with the notion that a genome duplication event occurred in the teleost lineage ∼350 million years ago, prior to the beginning of the teleost radiation [16], [17], [24]. While the majority of duplicated genes have been lost since the genome duplication event in the teleost lineage, a substantial percentage of the duplicates have been retained. When the putative zebrafish orthologs of 74 human genes on chromosome 17 were examined, Postlethwait et al. found that 15 (20%) are present as duplicate genes [25]. Like the mammals, the IGF signaling systems in teleosts are composed of IGF ligands, receptors, and IGFBPs [8], [15]. It has been shown previously that zebrafish contain two *igf-1r* genes and two insulin receptor genes [18]. Recent studies suggest that there are two *igf-1* genes and two *igf-2* genes in zebrafish (our unpublished data). Additionally, there is also evidence indicating that zebrafish have two *igfbp-1* genes [26]. In this study, we have shown that there are two *igfbp-2* and two *igfbp-5* genes in zebrafish. Therefore, it appears that all major components in the fish IGF signaling pathway are retained as duplicates. This high rate of gene retention in genes belonging to the IGF signaling pathway in the ray-fin fish is interesting. Unlike mammals, most teleosts do not reach a static adult size but continue to grow indeterminantly well beyond puberty. Given its central importance in growth regulation, it is possible that the retention of many duplicates of major players in the IGF signaling system may play an important role in the continuous growth pattern of the teleost.

The retention of two functional igfbp-2 genes in zebrafish also provided a unique opportunity to gain insights into how the IGFBP genes may have evolved through partitioning of ancestral functions. Subfunction partitioning can involve protein structural changes and has important functional and evolutionary consequences [17]. Taking advantage of the zebrafish model, we examined the spatial and temporal expression patterns and biological activities of the duplicated *igfbp-2* genes. Analysis of expression patterns for zebrafish *igfbp-2a* and *igfbp-2b* clearly reveal spatial and temporal subfunction partitioning. While zebrafish *igfbp-2b* is expressed throughout the entire early developmental stages in a ubiquitous fashion, *igfbp-2a* mRNA is not detected until 10 hpf in a highly tissue specific and dynamic manner. In the adult stage, these two duplicates are expressed in overlapping yet distinct tissues. While both *igfbp-2a* and *-2b* are highly expressed in the liver in both the adult male and female fish, only IGFBP-2a mRNA was detected in the brain. There appears to be a sexual dimorphism in their expression patterns. IGFBP-2a mRNA was also detected in the muscle and gonad in the male at low levels but not the female adult fish. In contrast, IGFBP-2b mRNA was detected in the gut, kidney, ovary, and muscle in adult female fish only. These gender specific expression patterns are interesting in light of the recent findings that male mice had 66% higher circulating IGFBP-2 levels compared with females [13]. While the duplicated zebrafish IGFBP-2 genes exhibit clear temporal and spatial partitioning in their expression, our previous and current biochemical analysis suggest that the duplicated *igfbp-2* genes have similar biological properties. Both zebrafish IGFBP-2a and -b are secreted proteins that are capable of IGF binding. Furthermore, overexpression of zebrafish IGFBP-2a and -2b in zebrafish embryos had nearly identical phenotypes, i.e., they caused a similar decrease in the growth and developmental rates. These data suggest that the duplicated *igfbp-2* genes encode two functionally equivalent proteins but have evolved distinct spatial and temporal expression patterns.

A number of *in vivo* studies using transgenic mice have shown that IGFBP-2 has a predominantly inhibitory effect on IGF actions [4]. Our functional analysis using transgenic zebrafish suggests that the inhibitory action of IGFBP-2 is conserved across species. When overexpressed in developing zebrafish embryos, human IGFBP-2 had a growth inhibitory effect similar to that of zebrafish IGFBP-2. While the inhibitory effect of IGFBP-2 can be explained by its competition for IGF binding with the IGF-1R based on biochemical and cell culture studies [2], [4], [5], *in vivo* evidence supporting this theory is still scarce. In this study, we have tested this model using transgenic zebrafish embryos. Our data shows that mutation of the major IGF binding site of human IGFBP-2 completely abolished its biological activity in this *in vivo* animal model. These results have provided direct evidence that IGFBP-2 is a conserved regulatory protein and it acts primarily by binding to and inhibiting IGF actions during early development *in vivo*.

IGF-independent actions of IGFBP-2 have been documented in cultured mammalian cells [2], [4]. It has been suggested that an RGD motif in human IGFBP-2 may be partially responsible for its cell surface association. The RGD motif in human IGFBP-2 has been reported to be involved in binding to integrin and this interaction is important for IGFBP-2-induced SNB19 glioma cell migration [23]. Since all known vertebrate IGFBP-2s, including the zebrafish IGFBP-2b reported in this study, have an RGD motif in their C-domains, we tested the possible role of RGD in mediating any biological actions of IGFBP-2 in transgenic zebrafish *in vivo*. We discovered that mutation of the RGD motif in human IGFBP-2 did not cause any notable changes in the growth inhibitory effects of IGFBP-2 in this animal model. This is consistent with the report by [11], which showed that overexpression of an RGE IGFBP-2 mutant did not affect IGFBP-2 cell surface association in transgenic mice.

In addition to the RGD motif, human IGFBP-2 possesses a putative heparin-binding motif (HBD). Recent studies have suggested that the association of IGFBP-2 with the cell surface or the extracellular matrix is due to its ability to bind to proteoglycans [22], but the relative contributions of the HBD *in vivo* is unknown. Taking advantage of the zebrafish model and the fact that the biological action of human IGFBP-2 is conserved, we investigated the role of the HBD *in vivo*. Our results have shown that transgenic zebrafish overexpressing the HBDm were essentially indistinguishable from those overexpressing native human IGFBP-2, indicating that the mutation of the HBD does not alter the growth inhibitory action of IGFBP-2. This conclusion is further supported by the fact that the two zebrafish IGFBP-2s had similar biological effects even though the HBD motif is not present in these proteins [27].

In conclusion, we have shown that there are two functional IGFBP-2 genes in zebrafish and other teleosts, which likely resulted from a genome wide duplication event before the beginning of the teleost radiation. Both zebrafish *igfbp-2a* and *-2b* encode secreted proteins that bind IGFs. Gene expression and functional analysis suggest that the two fish IGFBP-2 genes have undergone subfunctionalization partitioning by evolving distinct gene expression patterns, while their protein functionality remains largely unchanged. We further show that IGFBP-2 is a conserved regulatory protein and it inhibits embryo growth and development primarily by binding to IGF and inhibiting its actions *in vivo*. This study has not only provided novel insights into the functional evolution of IGFBPs but also demonstrated the utility of the zebrafish model system in the study of IGFBP physiology.

## Materials and Methods

### Chemicals

Reagents and chemicals were purchased from Fischer Scientific (Pittsburgh, PA) unless noted otherwise. RNase-free DNase and restriction enzymes were obtained from Promega (Madison, WI) and New England Biolabs (Beverly, MA). *Pfu Turbo* DNA polymerase was from Stratagene (La Jolla, CA). Superscript II reverse transcriptase (RT) and oligonucleotide primers were obtained from Invitrogen Life Technologies, Inc. (Carlsbad, CA).

### Experimental animals

Zebrafish (*Danio rerio*) were maintained on a 14 h light/10 h dark cycle. Fertilized eggs, obtained by natural cross, were raised in embryo medium at 28.5°C and staged according to Kimmel et al. [28]. For *in situ* hybridization analysis, embryo medium was supplemented with 0.003% (w/v) 2-phenylthiourea to inhibit embryo pigment formation. All experiments were conducted in accordance with the guidelines approved by the University Committee on the Use and Care of Animals at the University of Michigan.

### Molecular cloning and physical mapping

Using the human IGFBP-2 and zebrafish IGFBP-2 amino acid sequence as queries, we searched the zebrafish genome database (http://www.ensembl.org/Danio_rerio/blastview), the medaka genome database (http://www.ensembl.org/Oryzias_latipes/blastview), the stickleback genome database (http://www.ensembl.org/Gasterosteus_aculeatus/blastview), the tetraodon genome database (http://www.ensembl.org/Tetraodon_nigroviridis/blastview), and the Fugu genome database (http://www.ensembl.org/Takifugu_rubripes/blastview). We found an additional zebrafish IGFBP-2 gene (ENSDARP00000019643), two medaka IGFBP-2 genes (ENSORLG00000014695 and ENSORLG00000002232), two stickleback IGFBP-2 (ENSGACG00000014280 and ENSGACG00000002506), two tetraodon IGFBP-2 genes (GSTENG00016851001 and GSTENG00015886001) and two fugu IGFBP-2 genes (ENSTRUG00000013702 And ENSTRUG00000003825). The full-length sequence of the new zebrafish IGFBP-2 was determined by 5′- and 3′- rapid amplification of cDNA ends (RACE) using the SMART RACE kit (Clontech, Mountain View, CA). Amino acid sequences of IGFBP-2s were aligned by CLUSTAL X. Phylogenetic analysis was done using full-length amino acid sequences by Neighbor-joining method in the MEGA4 program with protein Poisson distances. Gap sites in the alignment were not used in the phylogenetic reconstruction. The reliability of the estimated tree was evaluated by the bootstrap method with 1000 pseudo-replications. Synteny analysis was carried out based on Danio rerio Zv6 and Homo sapiens Build 36.3 (http://www.ensembl.org/Homo_sapiens/index.html)and data from zebrafish and human synteny map. Genomic structure was determined by searching the zebrafish genome (http://www.ensembl.org/Danio_rerio/index.html).

### Reverse transcription (RT)-PCR and whole mount in situ hybridization

Total RNA was isolated from adult zebrafish tissues and whole embryos using TRIzol reagent (Invitrogen Life Technologies, Inc., Carlsbad, CA) following the manufacturer's instruction. One µg of RNA was reverse-transcribed to single strand cDNA using SuperScript II reverse transcriptase according to the manufacturer's instructions. RT-PCR was performed with two sets of primers (IGFBP-2a: 5′- CGATGTTGTCCTATGTGAGT -3′ and 5′- GATCACCTCTTATCAGTGGA-3′ IGFBP-2b: 5′- GCATGTCTCTTGCATTGCTC -3′ and 5′- CGGACGAGGGTATCTGGACA-3′) using Taq-DNA polymerase with ornithine decarboxylase (odc) mRNA as internal control as reported previously (21).

For whole mount *in situ* hybridization analysis, plasmids encoding zebrafish IGFBP-2a 3′ UTR and IGFBP-2b partial ORF with 3′UTR cDNAs or other genes were linearized by restriction enzyme digestion, followed by *in vitro* transcription reactions with either T7 or SP6 RNA polymerase, and then generated as digoxigenin (DIG)-labeled RNA probes. The specificity of each of the IGFBP-2 riboprobe was verified by dot-blot analysis and shown not to cross-react with each other's target. Hybridization was carried out as described previously [18]. Images were captured with a Nikon DC50NN camera mounted to a Nikon Eclipse E600 microscope (Melville, NY).

### Construction of expression plasmids

To produce recombinant IGFBP-2 proteins for biochemical analysis, cDNAs encoding ORFs for zebrafish IGFBP-2b and human IGFBP-2 were amplified by PCR with restriction enzyme sites and Kozak sequence containing primers (zebrafish IGFBP-2b: forward 5′-TCCTCTCGAGCCACCATGTCTCTTGCATTGCTCTGCAGTTTG-3′; reverse 5′-TGTGAAGCTTTTTCTGCTGGGCGGTGGGTGGTTCCAGT-3′; human IGFBP-2: forward 5′-TCCTCTCGAGCCACCATGCTGCCGAGAGTGGGCTG-3′, and reverse 5′-TGTGAAGCTTCTGCATCCGCTGGGTGTGCA-3′). The amplified cDNA was subcloned into the pcDNA3.1/ Myc-His(-)A expression vector (Invitrogen).

For *in vivo* functional studies, zebrafish IGFBP-2a expression constructs were engineered. First, DNA fragments containing the entire ORF of zebrafish IGFBP-2a in which the stop codon was mutated were generated by PCR (forward 5′-TCCTCTCGA GCCACCATGTTGTCCTATGTGAGTTGCGGCT-3′; and reverse 5′-TGTGAAGCTT GTTTGGAGGGTCGACAGATGGATC-3′) and subcloned into the pcDNA3.1/ Myc-His(-)A construct using the XhoI and Hind III sites. To determine the importance of the ligand binding domain (LBD), heparin binding domain (HBD), and the RGD motif of IGFBP-2, three mutants (LBDm forward 5′-TCCTCTCGAGCCACCATGCTGCCGAGAGTGGGCTG-3′and reverse 5′-TGTGAAGCTTCTTGTCACAGTTGGGGATGTGC-3′, HBDm forward 5′- CCTGGAGGAGCCCAACAACCTGGCACCACCCCCTGCC-3′ and reverse 5′-GGCAGGGGGTGGTGCCAGGTTGTTGGGCTCCTCCAGG-3′, and RGDm forward 5′-CCACCATCCGGGGGGAACCCGAGTG-3′and reverse5′- CACTCGGGTTCCCCCCGGATGGTGG-3′) were generated by PCR using Pfu Turbo DNA Polymerase (Stratagene, La Jolla, CA) according to published sequences [6], [22], [23]. For this pcDNA3.1-hIGFBP-2 was used as template. The LBDm (truncated IGFBP2) was generated by the PCR method. For HBDm and RGDm, 0.25 mM of each PCR primer, 2 mM of dNTP, 1.25 units of Pfu Turbo DNA Polymerase, and 1×Cloned Pfu DNA Polymerase Reaction Buffer with 5% DMSO were used to mutagenize the template DNA by PCR (95°C 1′; 95°C 50″, 55°C 50″, 68°C 7′, for 18 cycles; 68°C 7′) in a total volume of 50 µl. Each reaction was incubated with 10 units of Dpn I at 37°C for one hour. The sequences were confirmed by T7 sequencing primer and a reverse primer at downstream of multiple cloning sites of pcDNA3.1/ Myc-His (-) A. All plasmids were sequenced at the University of Michigan DNA Sequencing Core Facility.

### Expression of recombinant IGFBP-2 proteins

Recombinant *c-Myc*- and 6xHistidine-tagged human and zebrafish IGFBP-2b were produced by transfecting the expression plasmids into human embryonic kidney (HEK) 293T cells. Cells were cultured in high glucose Dulbecco's modified Eagle's medium with 10% fetal bovine serum. The transfected cells were maintained with culture medium containing 10% FBS and antibiotics (100 units/ml Penicillin and 100 µg/mL streptomycin sulfate, Gibco BRL). After the cells reached confluence, they were incubated with serum free media (SFM) for another 48 h. The SFM containing recombinant proteins was harvested, and the protein expression was confirmed by Western immunoblotting and ligand blotting as described below.

### Western immunoblotting and ligand blotting analysis

The SFM containing recombinant proteins was separated by SDS-PAGE and transferred to Immobilon P membranes (Millipore Corp., Bedford, MA) and subjected to Western blot using monoclonal anti-*c*-*myc* (clone 9E10/SC40; Santa Cruz, CA) as reported previously. Ligand blot analysis was performed using digoxigenin-labeled human IGF-I following published procedure [29].

### Microinjection experiment

Capped mRNA synthesis was carried out using a commercial kit and linearized plasmid DNA as template (mMESSAGE mMACHINE kit; Ambion, Inc.). mRNA (800 pg per embryo) was microinjected into zebrafish embryos at the 1–2 cell stage as reported previously (14). GFP mRNA injected and wild type embryos were used as controls. After injection, embryos were placed in embryo rearing medium [30] and kept at 28.5°C. Body length and somite number were measured at 24 hpf as described previously [31].

Statistics- Values are means±S.E.M. Differences among groups were analyzed by one way ANOVA followed by Tukey's Multiple Comparison Test using GraphPad Prism version 5.00 (San Diego, CA). Significance was accepted at p<0.05.
